# Neuropsychiatric outcomes following strokes involving the cerebellum: a retrospective cohort study

**DOI:** 10.3389/fnins.2023.1203488

**Published:** 2023-07-03

**Authors:** Victoria A. Muller Ewald, Carolina Deifelt Streese, Joel E. Bruss, Kenneth Manzel, Lilian M. Montilla, Ilisa K. Gala, Daniel T. Tranel, Krystal L. Parker

**Affiliations:** ^1^Department of Psychiatry, The University of Iowa, Iowa City, IA, United States; ^2^Iowa Neuroscience Institute, The University of Iowa, Iowa City, IA, United States; ^3^Department of Neurosurgery, The University of Iowa, Iowa City, IA, United States; ^5^Department of Pediatrics, The University of Iowa, Iowa City, IA, United States; ^4^Department of Neurology, The University of Iowa, Iowa City, IA, United States; ^6^Department of Psychological and Brain Sciences, The University of Iowa, Iowa City, IA, United States

**Keywords:** stroke, posterior cerebellar artery, basilar artery, grooved pegboard test, stroop test, Rey auditory verbal learning test (RAVLT)

## Abstract

**Introduction:**

Given the wide-ranging involvement of cerebellar activity in motor, cognitive, and affective functions, clinical outcomes resulting from cerebellar damage can be hard to predict. Cerebellar vascular accidents are rare, comprising less than 5% of strokes, yet this rare patient population could provide essential information to guide our understanding of cerebellar function.

**Methods:**

To gain insight into which domains are affected following cerebellar damage, we retrospectively examined neuropsychiatric performance following cerebellar vascular accidents in cases registered on a database of patients with focal brain injuries. Neuropsychiatric testing included assessment of cognitive (working memory, language processing, and perceptual reasoning), motor (eye movements and fine motor control), and affective (depression and anxiety) domains.

**Results:**

Results indicate that cerebellar vascular accidents are more common in men and starting in the 5th decade of life, in agreement with previous reports. Additionally, in our group of twenty-six patients, statistically significant performance alterations were not detected at the group level an average of 1.3 years following the vascular accident. Marginal decreases in performance were detected in the word and color sub-scales of the Stroop task, the Rey Auditory Verbal Learning Test, and the Lafayette Grooved Pegboard Test.

**Discussion:**

It is well established that the acute phase of cerebellar vascular accidents can be life-threatening, largely due to brainstem compression. In the chronic phase, our findings indicate that recovery of cognitive, emotional, and affective function is likely. However, a minority of individuals may suffer significant long-term performance impairments in motor coordination, verbal working memory, and/or linguistic processing.

## Introduction

**Figure 1:**
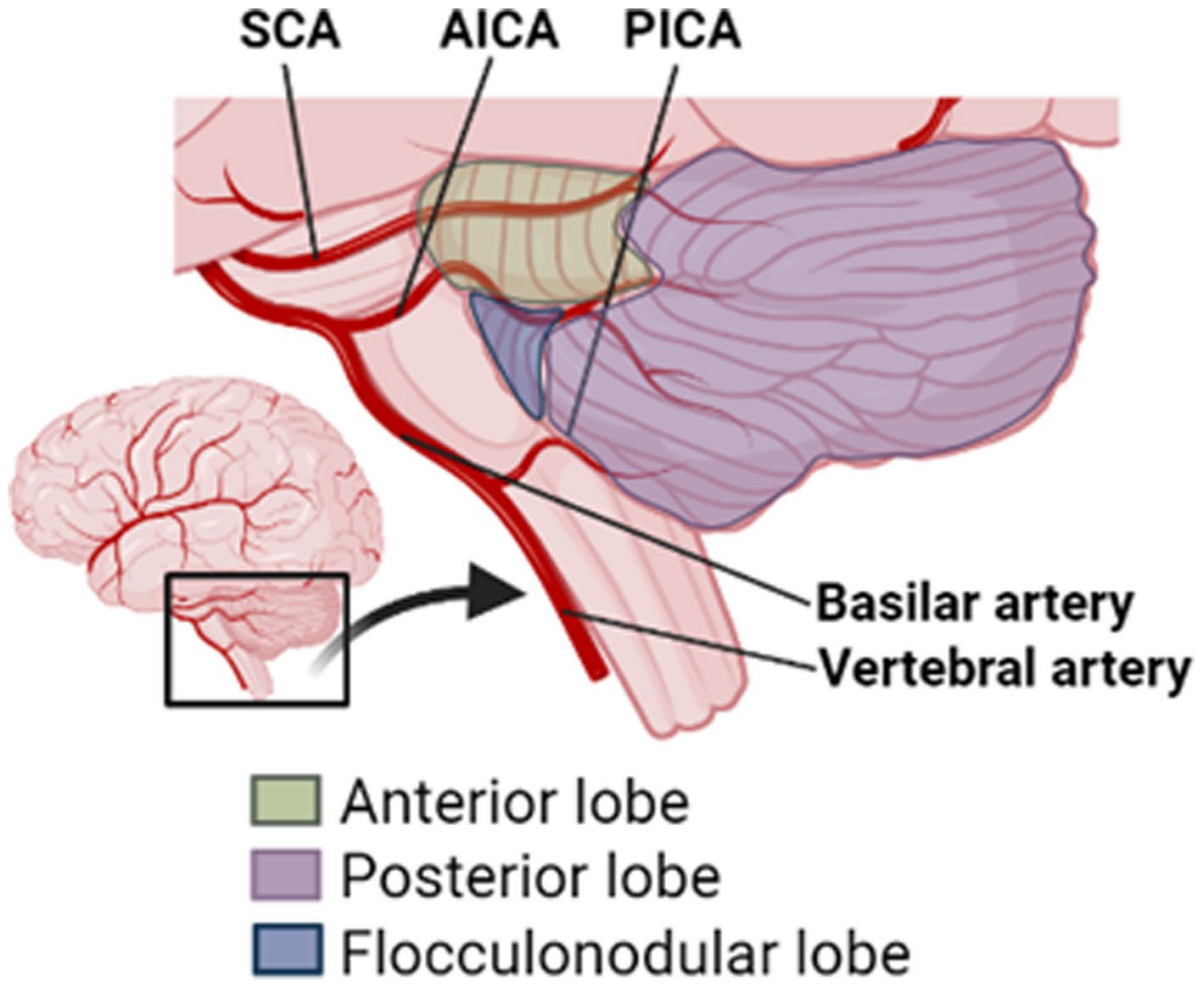
Schematic diagram of cerebellar lobules and arteries. SCA, superior cerebellar artery; AICA, anterior inferior cerebellar artery; and PICA, posterior inferior cerebellar artery.

Vascular accidents involving cerebellar blood supply are rare, thus outcomes associated with cerebellar strokes are understudied in comparison to cerebral strokes (Feely, 1979; Marinković et al., 1995; Edlow et al., 2008). Cerebellar ischemic strokes, usually caused by cardioembolism or large vessel atherosclerotic disease (Villalobos-Diaz et al., 2022), account for 2–3% of all ischemic strokes. Whereas cerebellar hemorrhagic strokes, mainly caused by vascular changes secondary to hypertension (Tsitsopoulos et al., 2011; Villalobos-Diaz et al., 2022), account for 9–10% of all intracranial hemorrhages (Villalobos-Diaz et al., 2022). The cerebellum receives blood supply from the posterior inferior cerebellar artery (PICA), the anterior inferior cerebellar artery (AICA), and the superior cerebellar artery (SCA), branches of the basilar artery (Figure 1). Work suggests that PICA strokes are most common, followed by SCA and AICA strokes (Edlow et al., 2008; Tsitsopoulos et al., 2011).

Risk factors for cerebellar vascular accidents include hypertension, diabetes, cigarette smoking, hyperlipidemia, and a history of stroke (Edlow et al., 2008). Cerebellar strokes occur more frequently in males than females, and are more prominent starting in the fifth decade of life (Villalobos-Diaz et al., 2022). Although cerebellar stroke symptoms can be severe, including coma and quadriplegia, such extremes are rare (Edlow et al., 2008). Common signs of cerebellar vascular accidents include drowsiness, dysarthria, dizziness/vertigo, headaches, oculomotor abnormalities, dysmetria, and limb ataxia (Feely, 1979; Patel and Zee, 2015). Dizziness and dysarthria are particularly common, presenting in more than half of patients (Edlow et al., 2008). Because cerebellar strokes present with common and non-specific symptoms, diagnosis can be challenging (Edlow et al., 2008). Additionally, CT scans, the most readily available brain imaging technique used to identify strokes, rarely identify early-stage cerebellar infarctions (Edlow et al., 2008). Thus, careful attention should be paid to patients’ motor coordination, gait, and eye movements – components of neurological examinations, which may be abridged if a cerebellar stroke is not being considered (Edlow et al., 2008).

Estimates of mortality rates associated with cerebellar strokes are considerably heterogeneous ranging from 14 to 56% (Neugebauer et al., 2013; Villalobos-Diaz et al., 2022). Because the posterior fossa provides little compensating space, space-occupying lesions can lead to life-threatening complications through brainstem compression (Neugebauer et al., 2013; Villalobos-Diaz et al., 2022). Upward herniation of the superior cerebellar vermis through the tentorial notch or downward herniation of the cerebellar tonsils through the foramen magnum are the most common causes of death in the acute phase following a cerebellar vascular accident (Neugebauer et al., 2013). Notably, the proximal branches of the main cerebellar arteries also typically supply blood to the lateral part of the brainstem. Therefore, coincident brainstem signs can be observed in patients with cerebellar stroke (Edlow et al., 2008).

It is common notion among neurosurgeons and neurologists that long-term outcomes are positive for patients who undergo successful life-saving interventions following cerebellar strokes (Neugebauer et al., 2013). However, few large studies have examined long-term (over 1 year) outcomes associated with cerebellar vascular accidents. Work by Tsitsopoulos et al. (2011) followed patients to 67 months post-stroke, reporting positive outcomes on the Modified Rankin Scale for Neurologic Disability for 77% of patients. Although this work addresses questions relevant to patients’ ability to regain function, it does not provide information on which *specific* neuropsychiatric domains were affected. Similarly, work by Feely (1979) included a large number of patients (75 cases). Cases were described as having made full or uneventful recoveries, and in some cases disability status of the patient was noted (“[…] she resumed her previous occupation as a housewife without any disability”). However, specific long-term neuropsychiatric outcomes were not assessed.

It is well established that cerebellar injury commonly leads to ataxia and/or difficulty with motor learning (Edlow et al., 2008). However, neuroanatomical, neuroimaging, and clinical studies have provided significant evidence to extend the cerebellum’s role into the modulation of cognitive and affective processing (Ivry and Keele, 1989; Andreasen et al., 1998; Schmahmann and Sherman, 1998; Stoodley, 2012; Stoodley, 2016). Indeed, although not the most prominent symptom following cerebellar vascular damage, deficits to cognitive functions including visuo-spatial planning, executive functions, and memory are reported in several case-studies (De Smet et al., 2011). Marien et al. (2007) described a patient who presented with apraxic agraphia, aphasia, dysexecutive symptoms, and behavioral and affective changes following a right cerebellar hemorrhagic lesion. Additionally, numerous reports detail patients who suffered cerebellar strokes and also presented with language impairments (De Smet et al., 2013). Karacı et al. (2008) investigated linguistic function in 20 patients with vascular damage to the cerebellum and found various aphasic symptoms including deficits in speech production, comprehension, repetition, naming, reading, and writing.

Beyond case reports, examinations of long-term neuropsychiatric functioning following cerebellar vascular accidents are lacking. Thus, the purpose of this study was to assess clinical characteristics and neuropsychiatric outcomes of a group of 26 patients who suffered cerebellar vascular accidents. The present clinical series is exploratory in nature; thus, results should be interpreted as revealing areas where future studies may focus.

## Methods

### Participants

A sub-set of cases from the Iowa Neurological Patient Registry, a large database of individuals with focal brain injuries, were retrospectively examined. The study period ranged from 1994 to 2022. Available data included demographic information, diagnosis, etiology, and performance on neuropsychiatric scales. Inclusion criteria for induction into the Iowa Neurological Patient Registry were: a clear and analyzable brain lesion that can be identified on magnetic resonance imaging (MRI), age 18 years or older, and ability to provide informed consent. Exclusion criteria were: evidence of preexisting dementia, significant cognitive impairment, severe psychiatric disease, and current or recent history of heavy alcohol or drug use. Inclusion criteria for the present study were: a diagnosis of cerebellar ischemic or hemorrhagic stroke. Patients with a diagnosis of epilepsy were excluded. All participants provided informed consent. Data collection was conducted with approval from the University of Iowa Institutional Review Board.

Cerebellar vascular accidents comprise a minority of all strokes, approximately 5%. Because such cases are uncommon, all Iowa Neurological Patient Registry cases of vascular accidents affecting the cerebellum were analyzed, including participants whose damage extended into other brain regions. Within the 38-year study period, 26 cases met inclusion criteria. Limitations of this study design are further addressed in the discussion. Participant characteristics are presented in Table 1.

**Table 1:** Table of patient characteristics.

Characteristic	Proportion of sample
Total sample size	26 (100%)
Stroke laterality	
Right	13 (50%)
Left	12 (46.2%)
Bilateral	1 (3.8%)
Self-reported sex	
Female	10 (38.5%)
Male	16 (61.5%)
Race	
Caucasian	25 (96%)
Unknown	1 (4%)
Ethnicity	
Hispanic	0 (0%)
Not Hispanic	19 (73%)
Unknown	7 (27%)
Years of education	13.9 (3.4)
Age of stroke onset	57.6 (15.2)
Chronicity	1.4 (1.8)
Damage to	
Cerebellum only	8 (30.8%)
Cerebellum and cortex	9 (34.6%)
Cerebellum and sub-cortex	3 (11.5%)
Cerebellum, cortex, and sub-cortex	6 (23.1%)

Discrete variables displayed as count (percent); continuous variables displayed as mean (standard deviation).

### Neuropsychological measures

In our neuropsychiatric battery, cognition was assessed via the Stroop Test (Stroop, 1935), Trail Making Test (TMT; Reitan, 1956), select subtests from the Wechsler Adult Intelligence Scale 4th Edition (Wechsler, 2008), Rey Auditory Verbal Learning Test (RAVLT; Rey, n.d.), Controlled Oral Word Association Test (COWAT; Al et al., 1997), and Rey-Osterrieth Complex Figure Test (CFT; Rey and Osterrieth, 1941). Motor performance was assessed via the Lafayette Grooved Pegboard Test (GPT; Matthews and Klove, 1964). Affect was assessed *via* the Beck Depression Inventory—II (BDI; Beck et al., 1996), and the Beck Anxiety Inventory (BAI; Steer and Beck, 1997). Due to time constraints or participant fatigue, not all participants completed all tests. A summary of tests completed is presented in Table 2.

**Table 2:** Tests administered in neuropsychiatric battery and domains assessed.

Measure	Number of patients	Domain assessed
Stroop		Selective attention, processing speed
Word	10 (38.5%)	Word reading
Color	10 (38.5%)	Color naming
Incongruent	10 (38.5%)	Interference and inhibition
TMT	21 (80.8%)	A: Visual search, B: set-shifting, attention
WAIS		Reasoning, processing speed, verbal comprehension
Similarities	11 (42.3%)	Abstract thinking
Vocabulary	9 (34.6%)	Language
Block design	11 (42.3%)	Visuospatial reasoning
Matrix reasoning	11 (42.3%)	Non-verbal reasoning
Digit span	12 (46.1%)	Working memory
Digit-symbol	12 (46.1%)	Processing speed, visual working memory
Symbol search	12 (46.1%)	Visuomotor processing speed
RAVLT		Verbal information encoding, storage and recovery
Trial 1	20 (76.9%)	Verbal working memory
Delayed recall	19 (73.1%)	Verbal memory
Delayed Recognition	19 (73.1%)	Verbal memory
COWAT	22 (84.6%)	Oral fluency
CFT	20 (76.9%)	Visuo-constructional ability, visual memory
GPT		Fine motor skills
Dominant	16 (61.5%)	
Non-dominant	14 (53.8%)	
BDI	16 (61.5%)	Depression
BAI	11 (42.3%)	Anxiety

Count (percent of total sample). TMT, trail making test; WAIS, Wechsler adult intelligence scale, RAVLT, Rey auditory verbal learning test; COWAT, controlled oral word association test; CFT, complex figure test; GPT, grooved pegboard test; BDI, beck depression inventory; and BAI, beck anxiety inventory.

For comparison to healthy populations, *z*-scores were calculated using population means and standard deviations from testing manuals. This transformation accounts for participant age, years of education, and, where appropriate, sex. Positive *z*-scores indicate better performance while negative *z*-scores indicate worse performance, compared to the neuronormative population. TMT and GPT scores, which indicate time-to-completion, were inverted for visualization purposes such that lower scores indicate poorer performance. Mood measures (BDI and BAI) are not converted into *z*-scores but rather reported as categories.

### Data analyses

Performance on neuropsychiatric measures was assessed in GraphPad Prism version 8.4.3 (GraphPad Software, San Diego, California, United States) using a one sample *t*-test. Multiple comparisons were corrected for using the Benjamini-Hochberg method. For evaluation of individual subjects’ performance, participants with *z*-scores below 2.8 (alpha = 0.05) were considered significantly impaired.

### Lesion quantification

To visualize lesion locations, lesions were manually traced on native brain scans in FSL (Jenkinson et al., 2012). The native MRI was then co-registered to the MNI152 1 mm atlas using non-linear registration with Advanced Normalization Tools (Avants et al., 2008). The spatial transformation warp was applied to the native lesion mask. The anatomical accuracy of each lesion mask was reviewed in both native and MNI space and edited as needed. Lesion volumes were calculated by multiplying the number of affected volumes by voxel volume (1 mm^3^).

Lesion subtractions were created to visualize areas, which were uniquely affected in individuals who suffered significant impairments following a vascular accident vs. those who did not. Subtractions were performed according to methods detailed in Rudrauf et al. (2008). In brief, for each neuropsychiatric test, separate lesion overlap maps were created for individuals who showed significant impairments vs. those who did not. Each lesion overlap map was divided by its own max-overlap in order to place all maps on the same proportional scale. The proportional lesion map for unimpaired individuals was then subtracted from the proportional lesion map for impaired individuals.

## Results

### Sample characteristics

Twenty-six cases of damage to the cerebellum were identified. Sixteen participants were male and 10 female. Age of vascular accident-onset ranged from 29 to 92 years with an average of 58 years. The average time between stroke-onset and neuropsychological testing was 1.37 years (SD 1.8 years). On average, participants were mildly depressed with a BDI score of 14.13, however, scores were considerably heterogeneous ranging from 0 (no depression) to 35 (severe depression). Anxiety scores were similarly heterogeneous ranging from 1 (minimal anxiety) to 31 (severe anxiety), with an average of 9 indicating mild anxiety.

### Vascular accident characteristics

**Figure 2:**
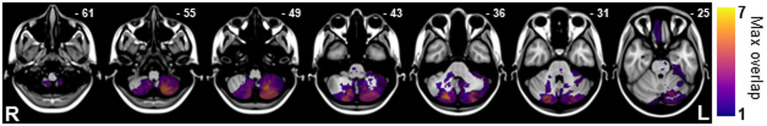
Overlay of lesions resulting from cerebellar vascular accidents. Maximum lesion overlap (*n* = 7) occurred in the right Crus II, followed by the left lobule VIIIa and a portion of lobule VIIIb (*n* = 6).

85% of cases (*n* = 22) were diagnosed with ischemic strokes, while 15% of cases (*n* = 4) were diagnosed with hemorrhagic strokes. 50% of cases (*n* = 13) suffered right cerebellar damage, 46% (*n* = 12) left cerebellar damage, and 5% (one case) bilateral cerebellar damage. For cases including notes on which sub-tentorial arteries were affected (*n* = 7), damage was most common to the vertebral artery (57%), followed by the posterior inferior cerebellar artery (28%). There was considerable heterogeneity in cerebellar lesion size, ranging from 261 to 30,382 mm^3^, with an average lesion size of 6,790 mm^3^. Maximum lesion overlap (*n* = 7) occurred in right crus II, followed by left lobule VIIIa and a portion of lobule VIIIb (*n* = 6; Figure 2). For individual lesion maps, see Supplementary Figure 1. For assessment of patients with legions restricted to the cerebellum see Supplementary material.

### Neuropsychiatric assessment—Group level

**Figure 3:**
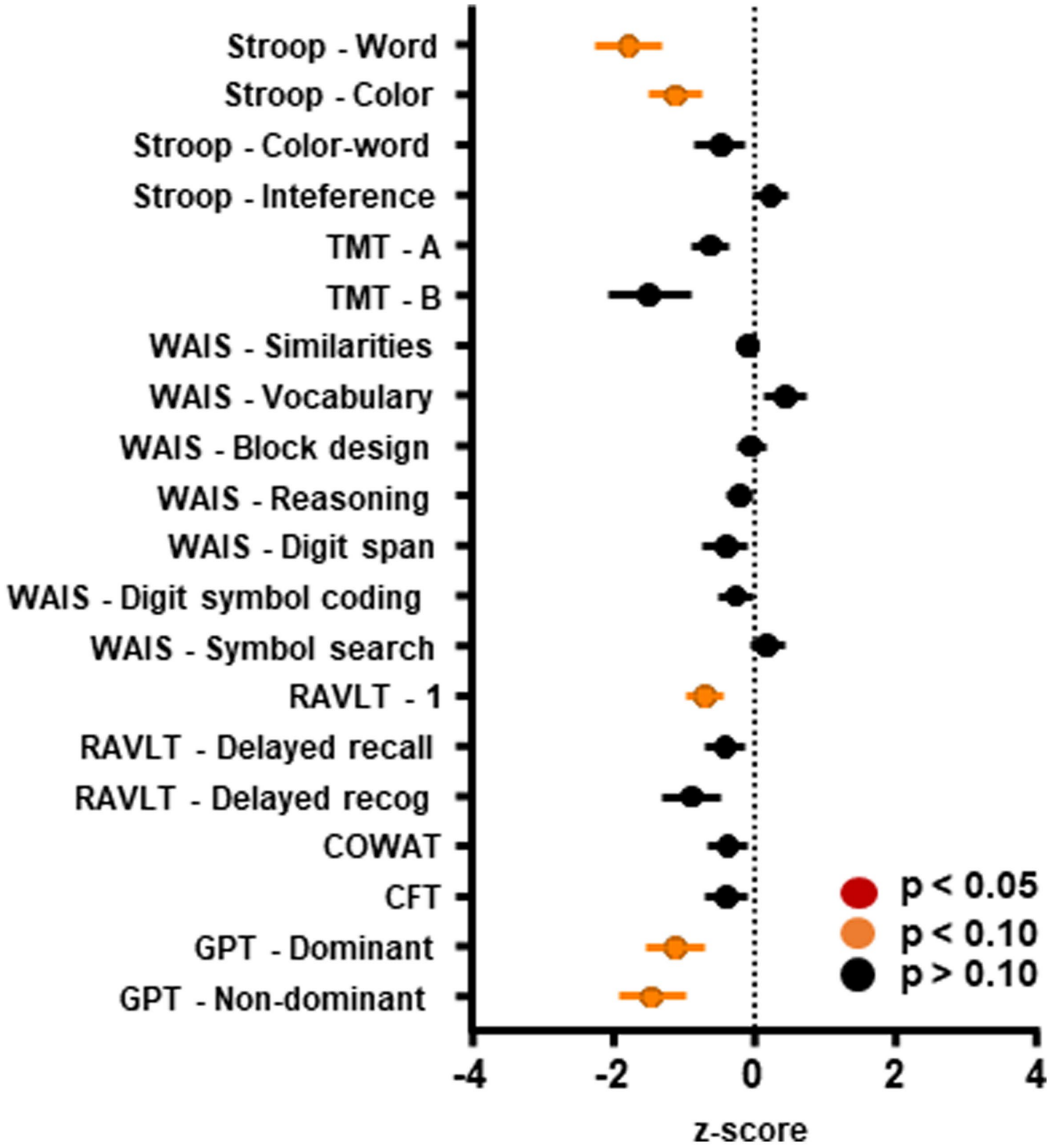
Performance on neuropsychiatric scales. Scores on neuropsychiatric scales administered to individuals who suffered vascular accidents involving the cerebellum. Marginal effects were detected such that cerebellar vascular damage led to worse performance on the Stroop task (word and color sub-scales), the first trial of the Rey Auditory Verbal Learning Test, and the Grooved Pegboard task. Raw scores have been Z-score transformed, where 0 represents no change from the neuronormative population, a positive score represents better performance and a negative score represents worse performance than the neuronormative population, respectively. Error bars represent standard error of the mean. TMT, trail making test; WAIS, Wechsler adult intelligence scale; RAVLT, Rey auditory verbal learning test; COWAT, controlled oral word association test; CFT, complex figure test; and GPT, grooved pegboard test.

The main goal of the present case-series was to evaluate the effects of strokes involving the cerebellum using a neuropsychiatric battery in the chronic (+1 year) phase following vascular accidents. Visual inspection of Figure 3 suggests that the highest average level of impairment was observed in Stroop—word (mean − 1.77; SD ± 1.45), GPT—non-dominant (mean − 1.44; SD ± 1.79), and trail making B (mean − 0.76; SD ± 1.46). See Table 3 for means and test statistics associated with each test.

**Table 3:** Result from one sample *t*-test.

Test	Mean (SEM)	Corrected value of *p*
Stroop		
Word	**−1.770 (0.4573)**	***t*(9) = 3.871, *p* < 0.10**
Color	**−1.120 (0.3732)**	***t*(9) = 3.001, *p* < 0.10**
Incongruent	−0.4700 (3.667)	*t*(9) = 1.282, *p* > 0.10
Interference	0.2400 (0.2414)	*t*(9) = 0.9943, *p* > 0.10
TMT		
A	0.6073 (0.2741)	*t*(20) = 2.216, *p* > 0.10
B	0.7613 (0.3340)	*t*(18) = 2.279, *p* > 0.10
WAIS		
Similarities	−0.0910 (0.5184)	*t*(10) = 0.5822, *p* > 0.10
Vocabulary	0.4444 (0.2992)	*t*(8) = 1.486, *p* > 0.10
Block design	−0.03027 (0.1982)	*t*(10) = 0.1527, *p* > 0.10
Reasoning	−0.1819 (0.1978)	*t*(10) = 0.9196, *p* > 0.10
Digit span	−0.3889 (0.3252)	*t*(11) = 0.1.196, *p* > 0.10
Digit symbol coding	−0.2500 (0.2326)	*t*(11) = 1.075, *p* > 0.10
Symbol search	0.1944 (0.2443)	*t*(11) = 0.7958, *p* > 0.10
RAVLT		
Trial 1	**−0.6830 (0.2704)**	***t*(19) = 2.526, *p* < 0.10**
Delayed recall	−0.4074 (0.2774)	*t*(18) = 1.468, *p* > 0.10
Delayed recognition	−0.8849 (0.4260)	*t*(18) = 2.078, *p* > 0.10
		
COWAT	−0.3650 (0.2986)	*t*(21) = 1.222, *p* > 0.10
CFT	−0.3783 (0.3095)	*t*(19) = 1.222, *p* > 0.10
GPT		
Dominant	**−1.112 (0.4247)**	***t*(15) = 2.619, *p* < 0.10**
Non-dominant	**−1.442 (0.4776)**	***t*(13) = 3.020, *p* < 0.10**

Bolding denotes marginal differences (*p* < 0.10). Mean (SEM). *t*(degrees of freedom) = test statistic, *p* value. TMT, trail making test; WAIS, Wechsler adult intelligence scale, RAVLT, Rey auditory verbal learning test; COWAT, controlled oral word association test; CFT, complex figure test; and GPT, grooved pegboard test.

Test statistics were examined for significance using an alpha of 0.05; however, no significant effects were found. Due to the exploratory nature of these analyses, we also assessed the data for marginal effects, assuming an alpha of 0.10. Compared to neuronormative controls, patients showed marginally impaired performance in Stroop word [*t*(9) = 3.871, *p* = 0.0760], Stroop color [*t*(9) = 3.001, *p* = 0.0832], RAVLT trial 1 [*t*(19) = 2.526, *p* = 0.0824], GPT—dominant [*t*(15) = 2.619, *p* = 0.0824], and GPT—non-dominant [*t*(13) = 3.020, *p* = 0.0824]. Performance on the neuropsychiatric battery did not differ by side of lesion or chronicity (data not shown).

### Neuropsychiatric assessment—Individual level

To more closely examine scales where marginal effects were detected, we next examined individual performance within each scale. Significant impairment for an individual was defined as a *z*-score of −2.8 or below, representing two standard deviations below the group mean. For GPT—dominant, four patients (23%) showed significantly impaired scores (Table 4). This group included individuals with strokes in the cerebellum only (50%), and the cerebellum, cortex and sub-cortex (50%). For GPT—non-dominant, two patients (13%) showed significantly impaired performance (Table 5). This group included patients from the cerebellum & cortex lesion group (50%) and the cerebellum, cortex & sub-cortex lesion group (50%).

**Table 4:** Patients’ performance on the Grooved pegboard—dominant task in *z*-score.

Stroke location	*Z*-score
Cerebellum	−0.076
	0.626
	**−3.958**
	−1.739
	−0.308
	−0.414
	0.117
	**−3.439**
Cerebellum and cortex	0.679
	−0.497
Cerebellum and sub-cortex	−2.048
	0.037
	−1.875
Cerebellum, cortex, and sub-cortex	**−3.118**
	0.465
	**−3.458**
	1.185

Organized by stroke location. Bolded values denote significant impairment.

**Table 5:** Patients’ performance on the Grooved pegboard—non-dominant task in z-score.

Stroke location	*Z*-score
Cerebellum	−0.083
	0.227
	−1.381
	0.637
	−0.980
	−0.980
	−0.803
Cerebellum and cortex	0.074
	**−5.880**
Cerebellum and sub-cortex	−2.521
	−0.613
	−2.648
Cerebellum, cortex, and sub-cortex	−1.414
	**−3.922**
	0.012

Organized by stroke location. Bolded values denote significant impairment.

For RAVLT trial 1, examination of single-patient *z*-scores revealed that no patients were classified as significantly impaired (Table 6). This suggests that the marginal effects detected on RAVLT trial 1 result from slight impairments to most patients’ performance, as opposed to significant impairments to a small sub-group of patients.

**Table 6:** Patients’ performance on the Rey Auditory Verbal Learning Test in *z*-score.

Stroke location	*Z*-score
Cerebellum	0
	1.938
	−2.118
	0.722
	0.500
	−1.375
	−0.125
	−1.813
Cerebellum and cortex	−0.563
	−1.188
	0.313
	−0.563
Cerebellum and sub-cortex	0.688
	−0.563
	0.688
	−0.563
	−0.941
Cerebellum, cortex, and sub-cortex	−2.188
	−0.313
	−1.88
	−2.438
	−2.611
	0.167

Organized by stroke location. Bolded values denote significant impairment.

Examination of single-subject scores for the baseline word-reading trial of the Stroop test (“Stroop—word”) reveals four patients (40%) with severe impairments (Table 7). Interestingly, all patients displaying severe impairments suffered from lesions confined to the cerebellum. Finally, examination of single-subject scores for the color naming trail of the Stroop test (“Stroop—color”) reveals one subject (10%) with significant impairment whose lesion was confined to the cerebellum (Table 8).

**Table 7:** Patients’ performance on Stroop—word in *z*-score.

Stroke location	*Z*-score
Cerebellum	**−3.5**
	**−3.1**
	0.6
	**−3.0**
	0.2
	**−3.0**
Cerebellum and sub-cortex	−1.0
	−2.2
	−0.9
Cerebellum, cortex & sub-cortex	−1.8

Organized by stroke location. Bolded values denote significant impairment.

**Table 8:** Patients’ performance on Stroop—color in *z*-score.

Stroke location	*Z*-score
Cerebellum	**−3.6**
	−1.2
	0.6
	−1.2
	−0.4
	−2.2
Cerebellum and sub-cortex	−0.7
	−1.7
	−0.7
Cerebellum, cortex, and sub-cortex	−0.1

Organized by stroke location. Bolded values denote significant impairment.

### Lesion proportion differences

**Figure 4:**
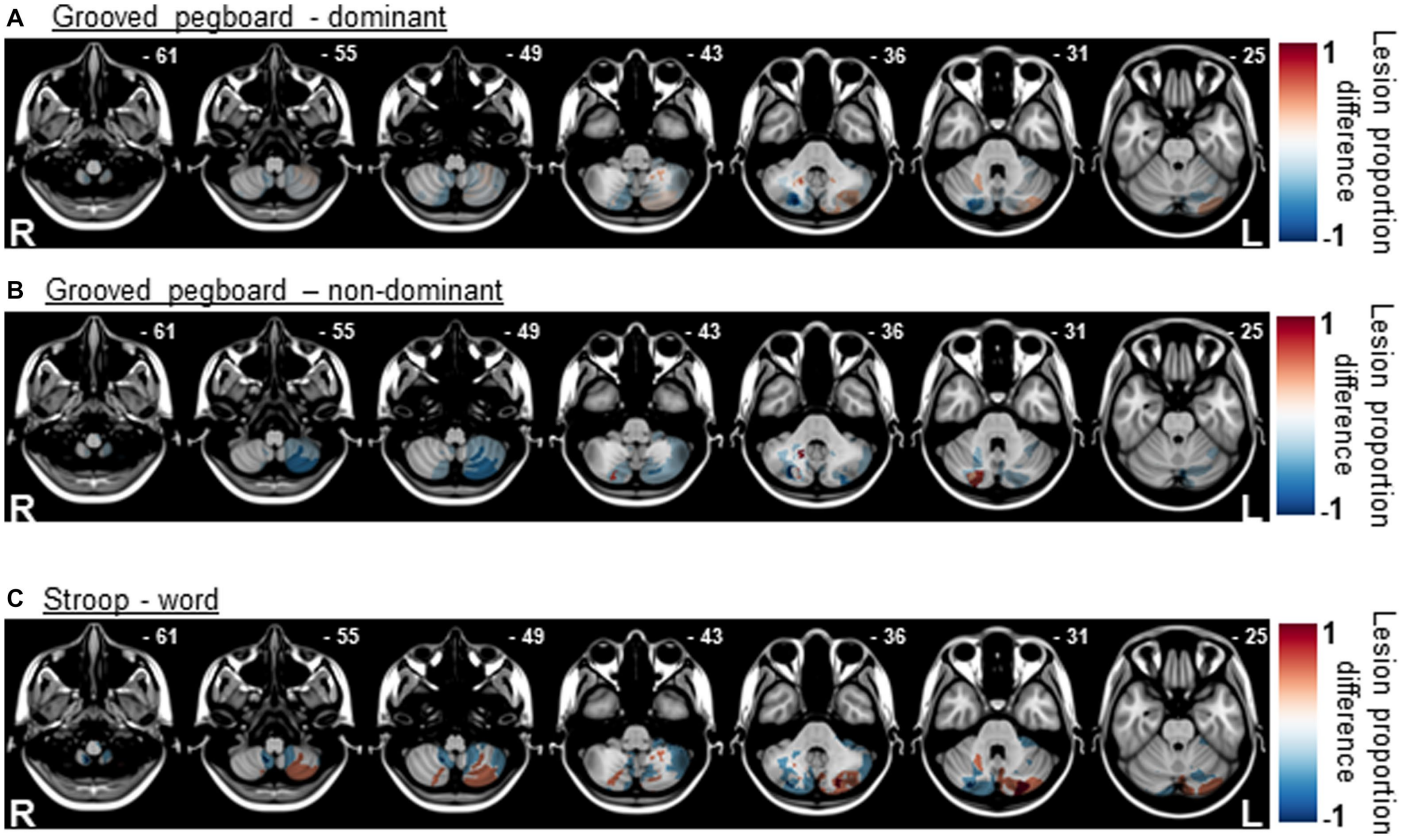
Lesion proportion difference maps for the Grooved Pegboard task **(A,B)** suggest little overlap in cerebellar regions by impairment classification. Lesion proportion difference map for Stroop—word **(C)**, suggest overlap in the crus I and lobule VII region for individuals who were significantly impaired on this task.

Lesion proportion difference maps were generated for scales where marginal impairments were detected to highlight cerebellar regions where damage may be associated with worsened performance. Results for the GPT (Figures 4A,B) suggest that there was little specificity/overlap for individuals who were significantly impaired in this task. Although lesions to crus II and lobules VII/VIII (*z* = −55 and − 49) were not associated with significant fine-motor impairments. Results for Stroop—word (Figure 4C) suggest overlap in the crus I and lobule VII region for individuals who were significantly impaired on this task. Because the “impaired” group consisted of zero individuals and a single individual for the RAVLT and Stroop—color tasks, respectively, lesion proportion differences maps were not generated for these tasks.

## Discussion

Sample demographics in the present clinical case-series aligned with previously published reports indicating that cerebellar vascular accidents are more prevalent among men (61.5%) and starting in the fifth decade of life (Edlow et al., 2008; Villalobos-Diaz et al., 2022). On a group level, cognitive, affective, and fine motor domains were not significantly impaired in the chronic stage (+1 year) following a cerebellar vascular accident. However, a minority of individuals suffered significant long-term impairments in motor coordination, verbal working memory, and/or linguistic processing, assessed via the GPT, RAVLT trial 1, and Stroop—word/color.

### Grooved peg-board task

In the present data-set, significant group-level differences in GPT performance were not detected. However, 23 and 13% of patients showed significant deficits in dominant and non-dominant hand performance on this task, respectively. Given the well-documented role of the cerebellum in motor coordination (Stoodley and Schmahmann, 2010; D'Angelo, 2018), deficits on the GPT, a measure of motor function, were expected. Indeed, impairments in GPT-dominant and non-dominant hand performance have previously been reported in spinocerebellar ataxia patients (Rentiya et al., 2017), a disorder marked by the degeneration of Purkinje and granule cells. Work suggests that GPT performance is related to the integrity of cerebellar medial superior regions, which are also commonly associated with motor task performance in fMRI studies (Stoodley, 2012; Rentiya et al., 2017). Given the central role of the cerebellum in motor coordination (D'Angelo, 2018), it is interesting that not all patients displayed deficits in this task. This is likely due to the chronicity associated with the vascular damage, on average more than 1-year post-stroke, which may have allowed for compensatory processes to occur.

### Stroop test

Impaired performance was observed on Stroop—color in a single patient, and on Stroop—word in four patients. Impairments in Stroop performance, specifically on color and word sub-tests, have been reported in cerebellar ataxia patients (Braga-Neto et al., 2012). Additionally, work suggests that patients with cerebellar lesions show impairments in reading words and sentences, making errors at the letter and the word level, a phenomenon known as cerebellar dyslexia (Moretti et al., 2002). Although the causes for this phenomenon are unclear, it may result from loss of eye-movement control, leading to binocular instability. Indeed, as the cerebellum significantly contributes to binocular alignment, cerebellar pathology often leads to nystagmus (Patel and Zee, 2015). However, it is also possible that reading difficulties associated with cerebellar damage might stem from disruption of normal communication between the cerebellum and the supra-tentorial regions implicated in linguistic processing. Indeed, tract-tracing work suggests connections between the right lateral cerebellum and the left frontal language areas (Turkeltaub et al., 2016).

Lesion subtraction results for Stroop—word suggest overlap in damage to Crus I and lobule VII in individuals who were significantly impaired on this task. This is in agreement with the larger literature suggesting that bilateral lobules VI and VII are involved in reading tasks (Stoodley, 2012). Activity in lobule VII is also reported in tasks, which tax verbal working memory. Cerebellar activation during such tasks may result from sub-vocal rehearsal, an important component of working memory (Krienen and Buckner, 2009). One interesting route for future work is to disentangle the mechanisms behind the reading deficits observed to determine if they are due to loss of articulatory control, eye movement difficulties, or cognitive dysmetria-like mechanisms (Andreasen et al., 1998). Indeed, all three mechanisms may play a role. Previous work suggests that language and reading are topographically organized within the cerebellum: while articulation engages anterior sensorimotor regions corresponding to articulatory muscles, verbal fluency/word generation engage posterolateral cognitive regions (Turkeltaub et al., 2016).

### Rey auditory verbal learning test

The marginal impairments observed in the present case-series on the RAVLT further suggest links between the cerebellum and language processing/production. The RAVLT is primarily a verbal test with the first trial specifically assessing attention and immediate memory. Unlike the other two tests on our neuropsychiatric battery where marginal impairments were detected, average RAVLT scores were not largely driven by a small group of significantly impaired patients. Instead, most patients showed slight impairments to their scores. This indicates that, although the association is small, cerebellar activity is important for verbal working memory, a notion supported by the broader literature.

### Symptom heterogeneity

It is worth mentioning two tests where impairments were surprisingly not detected in our cohort: the CFT and the TMT. The CFT assesses visuo-constructional ability and visual memory. Drawing complex figures relies on fine-motor coordination, visuospatial perception, spatial orientation, non-verbal memory, planning, and organization (Zhang et al., 2021). The involvement of the cerebellum in visuospatial abilities has been evidenced in numerous studies (Garcia et al., 2020; Zhang et al., 2021). Work suggests that individuals with cerebellar damage show worsened performance on tasks that involve mental imagery, mental object rotation, or visuocontructive demands (Garcia et al., 2020). Work by Olivito et al. (2018) found significant correlations between visuospatial performance impairment and gray matter loss in lobules V, VIIb, VIIIa, Crus I, Crus II, and the vermis. Additionally, our study did not confirm previous reports that patients with cerebellar damage show impairments on the TMT (Braga-Neto et al., 2012; Rentiya et al., 2017). The TMT is a robust assessment of visual search speed, visual attention, task switching, and mental flexibility. Work suggests that TMT performance-difficulties are associated with gray matter degeneration in the inferior hemispheric region of lobules VIIb and VIIIa.

An assessment of the broader literature suggests significant disagreement between works describing the effects of cerebellar damage, likely resulting from heterogeneous clinical presentations. A significant contributor to this heterogeneity is differences in etiology between works. Indeed, different presentations are expected, for example, between ataxias, which selectively damage specific cell types vs. strokes, which damage all cell types and fibers of passage. Lesion chronicity is another important contributor to symptom heterogeneity. While acute symptoms of cerebellar vascular accidents may be pronounced (Edlow et al., 2008); compensatory mechanisms can lead to a positive prognosis in the chronic phase. In the present work specifically, where the mean chronicity was 1.3 years, compensatory mechanisms may have taken place resulting in a masking of the association between vascular accident location and resulting symptoms. Finally, the heterogeneity of symptoms described in the literature might also be indicative of the wide-ranging domains in which the cerebellum is involved, including motor, cognitive and affective.

### Limitations

In addition to the caveats addressed above, our retrospective clinical case-series design also presents significant limitations. First, race/ethnicity were largely heterogeneous within our study population consisting almost entirely of Caucasian and non-Hispanic patients. This is a particularly significant caveat in the case of work relating to stroke, as studies suggest that factors relating to racial/ethnic disparities significantly impact outcomes (Skolarus et al., 2020). Thus, outside of Caucasian/non-Hispanic individuals we do not know how the characteristics we identify would extrapolate to the larger population. Additionally, although this design allowed for the assessment of rare occurrences, the number of samples was too small to identify a true association. Indeed, in our study period of 38 years, only eight cases consisting of damage *restricted* to the cerebellum were available. Cases including damage to other brain regions were thus included in our sample, introducing a potential none-differential misclassification bias, which could artificially deflate or inflate the associations observed in this study. Thus, it is prudent to interpret the results of the present case-series as revealing areas where future studies may focus.

## Conclusion

The present work is in agreement with the notion commonly held by clinicians that long-term cognitive outcomes following cerebellar vascular accidents are generally positive (Neugebauer et al., 2013). A small proportion of individuals showed long-term impairments in motor functioning and verbal skills as evidenced by impairments in the GPT, Stroop, and RAVLT. Although mechanisms underlying cerebellar associations with motor functioning are well described, future work should focus on uncovering mechanisms linking cerebellar damage to language and reading abilities.

## Data availability statement

The raw data supporting the conclusions of this article will be made available by the authors, without undue reservation.

## Ethics statement

The studies involving human participants were reviewed and approved by University of Iowa Hawk IRB. The patients/participants provided their written informed consent to participate in this study. Written informed consent was obtained from the individual(s) for the publication of any potentially identifiable images or data included in this article.

## Author contributions

VM, CD, DT, and KP contributed to the conception and design of the present work. CD and KM organized and acquired the data neuropsychiatric data. JB generated the lesion mapping data. VM, LM, and IG organized the data, performed the statistical analyses, and generated figures. VM, CD, JB, and KP contributed to the preparation of the manuscript. All authors contributed to the article and approved the submitted version.

## Funding

This work was supported by The National Institutes of Health (P50 MH094258 to DT), The Kiwanis Spastic Paralysis and Allied Diseases of the Central Nervous System Research Foundation (to DT), Nelli Ball Research Trust (KP), and Department of Psychiatry/Iowa Neuroscience Institute internal funds (KP).

## Conflict of interest

The authors declare that the research was conducted in the absence of any commercial or financial relationships that could be construed as a potential conflict of interest.

## Publisher’s note

All claims expressed in this article are solely those of the authors and do not necessarily represent those of their affiliated organizations, or those of the publisher, the editors and the reviewers. Any product that may be evaluated in this article, or claim that may be made by its manufacturer, is not guaranteed or endorsed by the publisher.

## References

[ref1] Al B., Des K., and Sivan A.B., Multilingual aphasia examination. AJA associates (1997).

[ref2] Andreasen N. C. Paradiso S. O'Leary D. S. (1998). "cognitive dysmetria" as an integrative theory of schizophrenia: a dysfunction in cortical-subcortical-cerebellar circuitry? Schizophr. Bull. 24, 203–218. doi: 10.1093/oxfordjournals.schbul.a033321 9613621

[ref3] Avants B. Epstein C. Grossman M. Gee J. (2008). Symmetric diffeomorphic image registration with cross-correlation: evaluating automated Labeling of elderly and neurodegenerative brain. Med. Image Anal. 12, 26–41. doi: 10.1016/j.media.2007.06.004 17659998 PMC2276735

[ref4] Beck A.T. Steer R.A. Brown G., Beck Depression Inventory–II (1996).

[ref5] Braga-Neto P. Pedroso J. L. Alessi H. Dutra L. A. Felício A. C. Minett T.. (2012). Cerebellar cognitive affective syndrome in Machado Joseph disease: core clinical features. Cerebellum 11, 549–556. doi: 10.1007/s12311-011-0318-6 21975858

[ref6] D'Angelo E. (2018). Physiology of the cerebellum. Handb. Clin. Neurol. 154, 85–108. doi: 10.1016/B978-0-444-63956-1.00006-0 29903454

[ref7] De Smet H. J. Engelborghs S. Paquier P. F. De Deyn P. P. Marien P. (2011). Cerebellar-induced apraxic agraphia: a review and three new cases. Brain Cogn. 76, 424–434. doi: 10.1016/j.bandc.2010.12.006 21507544

[ref8] De Smet H. J. Paquier P. Verhoeven J. Marien P. (2013). The cerebellum: its role in language and related cognitive and affective functions. Brain Lang. 127, 334–342. doi: 10.1016/j.bandl.2012.11.001 23333152

[ref9] Edlow J. A. Newman-Toker D. E. Savitz S. I. (2008). Diagnosis and initial management of cerebellar infarction. Lancet Neurol. 7, 951–964. doi: 10.1016/S1474-4422(08)70216-3 18848314

[ref10] Feely M. P. (1979). Cerebellar infarction. Neurosurgery 4, 7–11. 450221 10.1227/00006123-197901000-00003

[ref11] Garcia M. Lazaro E. Amayra I. Lopez-Paz J. F. Martinez O. Perez M.. (2020). Analysis of visuospatial abilities in Chiari malformation type I. Cerebellum 19, 6–15. doi: 10.1007/s12311-019-01056-y 31286383

[ref12] Ivry R. B. Keele S. W. (1989). Timing functions of the cerebellum. J. Cogn. Neurosci. 1, 136–152. doi: 10.1162/jocn.1989.1.2.136 23968462

[ref13] Jenkinson M. Beckmann C. F. Behrens T. E. Woolrich M. W. Smith S. M. (2012). NeuroImage 62, 782–790. doi: 10.1016/j.neuroimage.2011.09.015 21979382

[ref14] Karacı R. Öztürk Ş. Özbakır Ş. Cansaran N. (2008). Evaluation of language functions in acute cerebellar vascular diseases. J. Stroke Cerebrovasc. Dis. 17, 251–256. doi: 10.1016/j.jstrokecerebrovasdis.2008.02.009 18755402

[ref15] Krienen F. M. Buckner R. L. (2009). Segregated fronto-cerebellar circuits revealed by intrinsic functional connectivity. Cereb. Cortex 19, 2485–2497. doi: 10.1093/cercor/bhp135 19592571 PMC2742600

[ref16] Marien P. Verhoeven J. Brouns R. de Witte L. Dobbeleir A. de Deyn P. P. (2007). Apraxic agraphia following a right cerebellar hemorrhage. Neurology 69, 926–929. doi: 10.1212/01.wnl.0000267845.05041.41 17724298

[ref17] Marinković S. Kovacević M. Gibo H. Milisavljević M. Bumbasirević L. (1995). The anatomical basis for the cerebellar infarcts. Surg. Neurol. 44, 450–460. 8629230 10.1016/0090-3019(95)00195-6

[ref18] Matthews C.G. Klove K., Instruction Manual for the Adult Neuropsychology Test Battery. Madison, WI: University of Wisconsin Medical School (1964).

[ref19] Moretti R. Bava A. Torre P. Antonello R. M. Cazzato G. (2002). Reading errors in patients with cerebellar vermis lesions. J. Neurol. 249, 461–468. doi: 10.1007/s004150200040 11967654

[ref20] Neugebauer H. Witsch J. Zweckberger K. Juttler E. (2013). Space-occupying cerebellar infarction: complications, treatment, and outcome. Neurosurg. Focus. 34:E8. doi: 10.3171/2013.2.FOCUS12363 23634927

[ref21] Olivito G. Lupo M. Iacobacci C. Clausi S. Romano S. Masciullo M.. (2018). Structural cerebellar correlates of cognitive functions in spinocerebellar ataxia type 2. J. Neurol. 265, 597–606. doi: 10.1007/s00415-018-8738-6 29356974

[ref22] Patel V. R. Zee D. S. (2015). The cerebellum in eye movement control: nystagmus, coordinate frames and disconjugacy. Eye 29, 191–195. doi: 10.1038/eye.2014.271 25397778 PMC4330284

[ref23] Reitan R.M., Trail Making Test. Manual for Administration, Scoring, and Interpretation. (1956).

[ref24] Rentiya Z. Khan N. S. Ergun E. Ying S. H. Desmond J. E. (2017). Distinct cerebellar regions related to motor and cognitive performance in SCA6 patients. Neuropsychologia 107, 25–30. doi: 10.1016/j.neuropsychologia.2017.10.036 29100951 PMC5705404

[ref25] Rey A. (1984). Rey Auditory Verbal Learning Test (RAVLT). Paris, France: Presses universitaires de France

[ref26] Rey A. Osterrieth P.A.A., Rey-Osterrieth complex figure copying test. PsycTESTS Dataset (1941).

[ref27] Rudrauf D. Mehta S. Bruss J. Tranel D. Damasio H. Grabowski T. J. (2008). Thresholding lesion overlap difference maps: application to category-related naming and recognition deficits. NeuroImage 41, 970–984. doi: 10.1016/j.neuroimage.2007.12.033 18442925 PMC2582202

[ref28] Schmahmann J. D. Sherman J. C. (1998). The cerebellar cognitive affective syndrome. Brain 121, 561–579. doi: 10.1093/brain/121.4.561 9577385

[ref29] Skolarus L. E. Sharrief A. Gardener H. Jenkins C. Boden-Albala B. (2020). Considerations in addressing social determinants of health to reduce racial/ethnic disparities in stroke outcomes in the United States. Stroke 51, 3433–3439. doi: 10.1161/STROKEAHA.120.030426 33104471 PMC7732185

[ref30] Steer R.A. Beck A.T., Beck Anxiety Inventory, Evaluating Stress: A book of Resources. Lanham, MD, US: Scarecrow Education (1997), 23–40.

[ref31] Stoodley C. J. (2012). The cerebellum and cognition: evidence from functional imaging studies. Cerebellum 11, 352–365. doi: 10.1007/s12311-011-0260-7 21373864

[ref32] Stoodley C. J. (2016). The cerebellum and neurodevelopmental disorders. Cerebellum 15, 34–37. doi: 10.1007/s12311-015-0715-3 26298473 PMC4811332

[ref33] Stoodley C. J. Schmahmann J. D. (2010). Evidence for topographic organization in the cerebellum of motor control versus cognitive and affective processing. Cortex 46, 831–844. doi: 10.1016/j.cortex.2009.11.008 20152963 PMC2873095

[ref34] Stroop J. R. (1935). Studies of interference in serial verbal reactions. J. Exp. Psychol. 8, 643–662.

[ref35] Tsitsopoulos P. P. Tobieson L. Enblad P. Marklund N. (2011). Surgical treatment of patients with unilateral cerebellar infarcts: clinical outcome and prognostic factors. Acta Neurochir. 153, 2075–2083. doi: 10.1007/s00701-011-1120-4 21833781

[ref36] Turkeltaub P. E. Swears M. K. D’Mello A. M. Stoodley C. J. (2016). Cerebellar tDCS as a novel treatment for aphasia? Evidence from behavioral and resting-state functional connectivity data in healthy adults. Restor. Neurol. Neurosci. 34, 491–505. doi: 10.3233/RNN-150633 27232953 PMC5469248

[ref37] Villalobos-Diaz R. Ortiz-Llamas L. A. Rodriguez-Hernandez L. A. Flores-Vazquez J. G. Calva-Gonzalez M. Sangrador-Deitos M. V.. (2022). Characteristics and long-term outcome of cerebellar strokes in a single health Care Facility in Mexico. Cureus 14:e28993. doi: 10.7759/cureus.28993 36259000 PMC9573303

[ref38] Wechsler D., Wechsler adult intelligence scale--fourth edition. PsycTESTS Dataset (2008).

[ref39] Zhang X. Lv L. Min G. Wang Q. Zhao Y. Li Y. (2021). Overview of the complex figure test and its clinical application in neuropsychiatric disorders, including copying and recall. Front. Neurol. 12:680474. doi: 10.3389/fneur.2021.680474 34531812 PMC8438146

