# Supplementary material for: Neuropsychiatric outcomes following strokes involving the cerebellum: a retrospective cohort study
Source: Front Neurosci. 2023 Jul 3;17:1203488. doi: 10.3389/fnins.2023.1203488 (PMC10352988; doi:10.3389/fnins.2023.1203488)
Supplement: Supplementary file 1 [file Data_Sheet_1.pdf]

Supplemental materials for: Neuropsychiatric outcomes following strokes involving the cerebellum: a retrospective cohort study

Supplemental results

The Iowa Neurological Patient Registry was queried for patients who suffered vascular accidents involving damage to the cerebellum. This resulted in 26 cases, 8 which suffered damage to the cerebellum only, 9 which suffered damage to the cerebellum and the cortex, 3 which suffered damage to the cerebellum and the sub-cortex, and 6 which suffered damage to the cerebellum, the cortex and the sub-cortex (**Supplemental table 1**). Individual images of lesion center of gravity for each participant are shown in **Supplemental figure 1**.

Supplemental figure 2 shows performance on the neuropsychiatric battery for individuals who suffered damage to the cerebellum only. The highest average levels of impairment were observed in Stroop – word (mean -1.76; SD ± 1.77), trail making B (mean -1.44; SD ± 3.80), and grooved pegboard – dominant (mean -1.31; SD ± 1.80). However, no statistically significant differences in performance were detected between the cerebellar stroke group and comparison group, likely due to limitations in power.

Supplemental figure 3 shows lesion proportion difference maps for the Grooved Pegboard test dominant and non-dominant hands, focusing on areas of non-cerebellar overlapping damage. There is very little overlap in cortical regions that differ between impaired and non-impaired individuals in this task, however results suggest that individuals with damage to the anterior temporal lobe did not tend to present with impairments in the Grooved Pegboard non-dominant task. Note that because the "impaired" group consisted of no individuals with damage outside of the cerebellum for the Stroop – word task, no significantly impaired individuals for the Rey Auditory Verbal Learning test, and a single individual for the Stroop – color task, lesion proportion differences maps were not generated for these tasks.

Supplemental figures

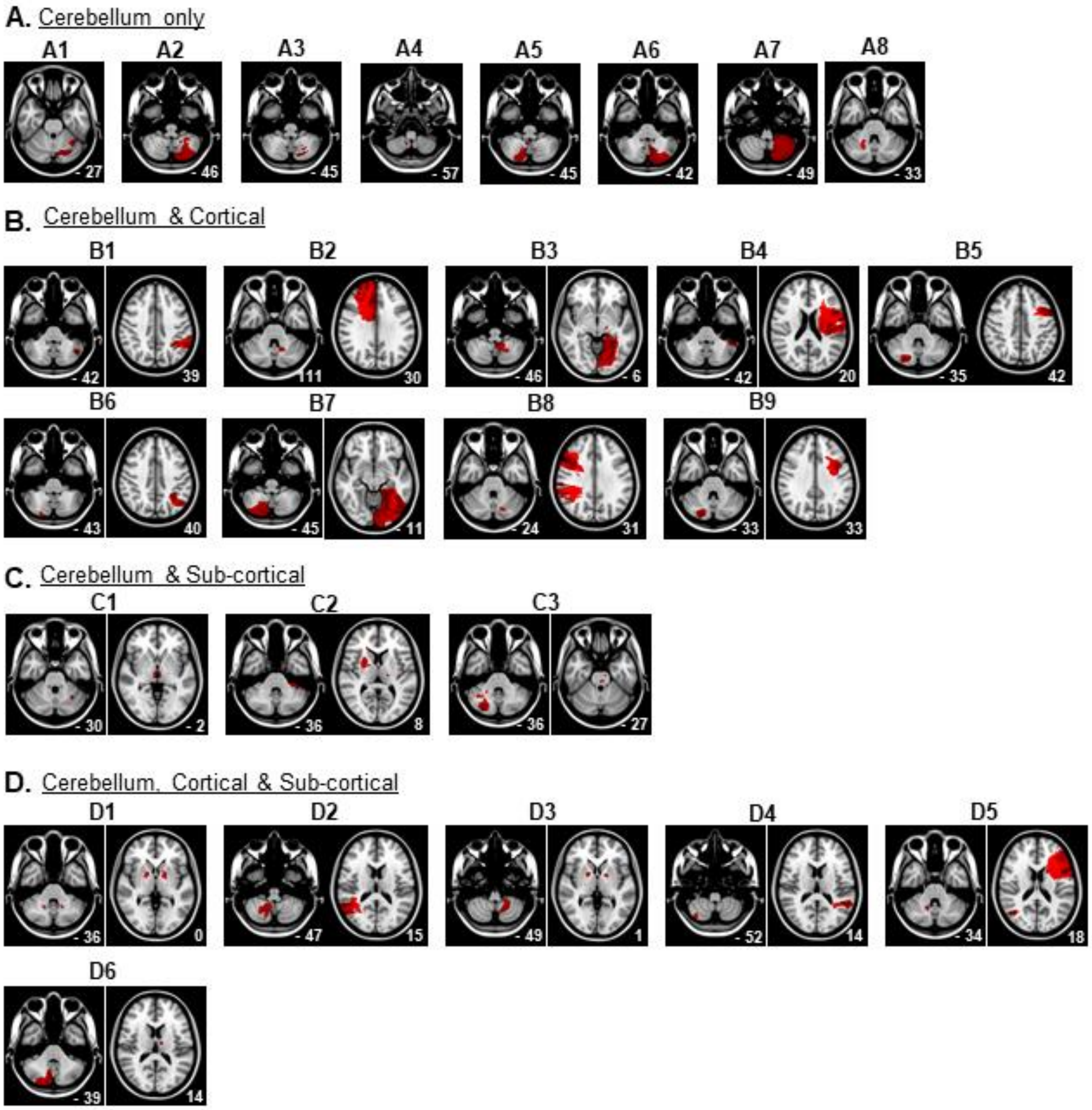

Supplemental figure 1. Individuals lesion maps for each case included in the present study sorted by lesion location group.

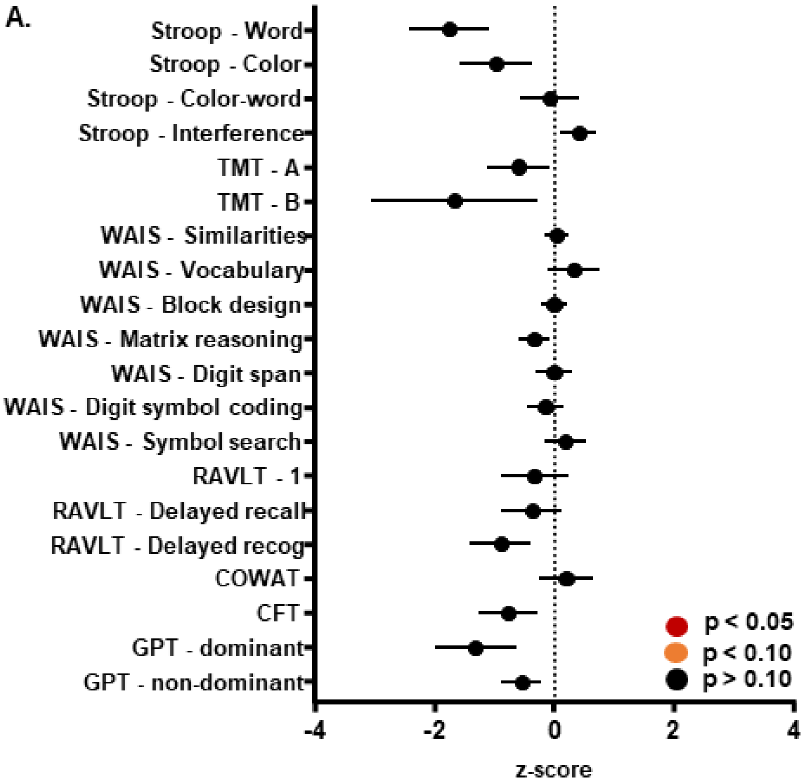

Supplemental figure 2. No significant alterations in task performance were observed for individuals who suffered damage to the cerebellum exclusively (n = 8). TMT, Trail Making Test; WAIS, Wechsler Adult Intelligence Scale; RVALT, Rey Auditory Verbal Learning Test; COWAT, Controlled Oral Word Association Test; CFT, Complex Figure Test; GPT, Grooved Pegboard Test.

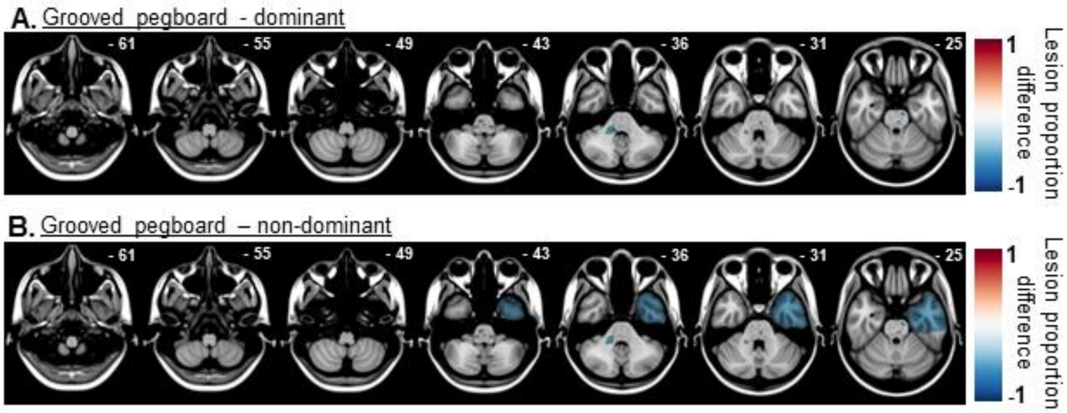

Supplemental figure 3. Lesion proportion difference maps for Grooved Pegboard – dominant (**A**) and non-dominant (**B**) tasks centered on cortical center of gravity.

Supplemental tables

Measure	Cerebellum	Cerebellum & cortex	Cerebellum & sub-cortex	Cerebellum, cortex & sub-cortex
Stroop	6 (23.08%)	0 (0%)	3 (11.5%)	1 (3.8%)
TMT	7 (26.9%)	5 (19.2%)	3 (11.5%)	6 (23.08%)
WAIS				
Similarities	6 (23.08%)	2 (7.7%)	0 (0%)	3 (11.5%)
Vocabulary	6 (23.08%)	2 (7.7%)	0 (0%)	1 (3.8%)
Block Design	7 (26.9%)	2 (7.7%)	0 (0%)	2 (7.7%)
Matrix Reasoning	7 (26.9%)	2 (7.7%)	0 (0%)	2 (7.7%)
Digit Span	7 (26.9%)	2 (7.7%)	0 (0%)	3 (11.5%)
Digit-Symbol Coding	7 (26.9%)	2 (7.7%)	0 (0%)	3 (11.5%)
Symbol Search	7 (26.9%)	2 (7.7%)	0 (0%)	3 (11.5%)
RAVLT				
Trial 1	7 (26.9%)	4 (15.4%)	3 (11.5%)	6 (23.08%)
Delayed Recall	6 (23.08%)	4 (15.4%)	3 (11.5%)	6 (23.08%)
Delayed Recognition	6 (23.08%)	4 (15.4%)	3 (11.5%)	6 (23.08%)
COWAT	7 (26.9%)	7 (26.9%)	3 (11.5%)	5 (19.2%)
CFT	7 (26.9%)	4 (15.4%)	3 (11.5%)	6 (23.08%)
GPT				
Dominant	7 (26.9%)	2 (7.7%)	3 (11.5%)	4 (15.4%)
Non-dominant	6 (23.08%)	2 (7.7%)	3 (11.5%)	3 (11.5%)
BDI	7 (26.9%)	5 (19.2%)	2 (7.7%)	2 (7.7%)
BAI	7 (26.9%)	2 (7.7%)	2 (7.7%)	2 (7.7%)

Supplemental table 1. Number of patients in each category who took each neuropsychiatric test. Number of individuals (Percent of total case count). TMT, trail making test; WAIS, Wechsler Adult Intelligence Scale, RAVLT, Rey Auditory Verbal Learning Test; COWAT, Controlled Oral Word Association Test; CFT, Complex Figure Test; GPT, Grooved Pegboard Test; BDI, Beck Depression Inventory; BAI, Beck Anxiety Inventory.
